# Effect of Window and Hole Pattern Cut-Outs on Design Optimization of 3D Printed Braces

**DOI:** 10.3389/fresc.2022.889905

**Published:** 2022-06-24

**Authors:** Robert Rizza, XueCheng Liu, Vince Anewenter

**Affiliations:** ^1^Department of Mechanical Engineering, Milwaukee School of Engineering, Milwaukee, WI, United States; ^2^Department of Orthopaedic Surgery, Milwaukee, WI, United States; ^3^Musculoskeletal Functional Assessment Center, Children's Hospital of Wisconsin, Milwaukee, WI, United States; ^4^Department of Orthopaedic Surgery, Medical College of Wisconsin, Milwaukee, WI, United States; ^5^Rapid Prototyping Center, Milwaukee School of Engineering, Milwaukee, WI, United States

**Keywords:** 3D printing, scoliosis braces, hole patterns, cut-outs, brace design

## Abstract

**Background:**

There are many different Thoracic Lumbar Sacral Orthosis style brace designs available in the market for the correction of scoliosis deformity. Hole cut out patterns, are commonly used in brace designs. These cut-outs may be subdivided into two groups: hole patterns and windows. Hole patterns are an array of holes which are implemented to lighten the weight of a brace and allow for the skin to breathe. Windows provide space for spinal derotation and/or breathing. From an examination of the literature, it appears that a systematic analysis of the effect of these cut-outs on the structural integrity and functionality of the brace has not been undertaken. Furthermore, there is a lack of understanding on the effect of spacing, size and geometry of the cut-outs on the mechanical behavior of the brace.

**Method of Approach:**

In this study, Finite Element Analysis is employed to examine the mechanical response of the brace to these cut-outs. Geometry for the Thoracic Lumbar Sacral Orthosis was obtained by scanning an existing brace using an optical scan and converted into a Computer Aided Design model. A systematic approach was undertaken where cut-out geometry, spacing and size was varied. The deformation and stress in the thickness of the brace was ascertained from the Finite Element Analysis. An appropriate factor of safety for the structural analysis was determined using a standardized approach and used to quantify the structural integrity of the brace due to the cut-out. Various geometries were analyzed for the hole patterns including circle, triangle, diamond, and hexagon. For the window, the geometries considered were circle, trapezoidal and the “bib” geometry.

**Results:**

It was found that linear hole patterns where the holes are aligned do not provide a desirable structural factor safety. Furthermore, among all the possible geometries, the hexagonal cut-out was the best structurally while reducing the weight of the brace the most. The optimal spacing was found to be 12 mm, and the optimal hole surface area was found to be 78.54 mm^2^. For the windows in the abdominal area, the “bib” shape provided the best structural integrity and generated the lowest amount of deformation. An increase in the size of this window had a small effect on the stress but an almost negligible effect on the deformation.

**Conclusions:**

A hexagonal hole pattern should be used with a spacing of 12 mm and each hole should have a surface area of 78.54 mm^2^. Windows in the abdominal area should be of “bib” shape. The size of the window cut-outs does not affect the brace stress and deformation significantly. Thus, the size of these windows should be based on the functional aspects of the brace, i.e., the minimum required size needed to permit the patient to breathe comfortably as in the case of the abdominal window or to allow for proper derotation, as in the case of the derotation window.

## Introduction

The prevalence of adolescent idiopathic scoliosis (AIS) is estimated from 2 to 3% for children between 10 and 16 years of age (1). With this disability not only does a larger spinal curve affect posture and cosmetics but it causes pain and pulmonary function and activity of daily life deficits.

The Thoracic Lumbar Sacral Orthosis (TLSO) is widely applied for Scoliosis and provides control of flexion, extension, lateral bending using a three-point loading mechanics and circumferential compression. A design by Thometz and Liu (2, 3) allows for more de-rotation. TLSO can be designed in modular form, including anterior and posterior polymer lateral panels (typically made of Polyethylene), padded with foam, and secured with Velcro straps. Some new designs incorporate 3D printing for the lateral panels (but not the padding) (4–7). By exploiting the accuracy and speed of the process, some advancements in brace design and manufacturing have been made.

TLSO are traditionally designed and manufactured using a hands-on process which is highly dependent on the experience level of the orthotist. In recent years, there has been much effort to implement Computer Aided Modeling (CAM) to design the brace (8). Some of these implementations make use of Finite Element Analysis (FEA) in the design process (9–13). With FEA, the spine, rib cage and torso are often modeled but not all FEA approaches completely model the torso and the impact from using FEA varies (14). Other methods make use of a classification system where the deformity is categorized by examining the pattern of the spine curve (Lehnert-Schroth Classification System) (15). This method, however, is not universally accepted and is limited by proper implementation of the classification system.

Often, small hole patterns are placed in the hard-shell elements of the brace to lighten the weight of the brace. These holes have the added beneficial property of preventing absorption of moisture. Moisture leads to odor, mildew growth and in some cases Dermatitis (16). In practice, there are many hole shapes that are used. These shapes include circle, triangle, diamond, and hexagonal geometries. Obviously, the greater the hole volume, the lighter the brace and the more surface area to permit breathing of the skin. However, as the size of the hole increases there is a greater impact on the structural integrity of the brace. A review of the literature suggests that no systematic investigation of the effect of these hole patterns on the structural integrity of the brace has been carried out. Nor has the effect of hole size or hole spacing been addressed.

In addition, cut-outs commonly referred to as a window are often used. A window is implemented in the abdominal area of the brace so that young children who breathe with their abdomen can breathe normally. A window is also implemented posteriorly and laterally on the brace to allow for derotation of the spinal column. As with the hole patterns, no systematic investigation has been undertaken to investigate optimal size of these windows or to examine the effect of their placement on the structural integrity of the brace.

The objective of the study described in this paper was to systematically investigate the use of hole patterns and windows on the structural integrity of the brace. The effect of these brace features on the design of the brace relative to patient comfort was also considered through direct engagement with practicing orthotists (4).

## Methods

### Brace Geometry, Mechanical Forces and Basic Brace FEA

A spine model was created using geometry of the spine and mechanical properties of the spinal elements. This data was taken from the literature (17). A finite element model was constructed of the spine [vertebrae: E=10 GPa, ν = 0.29, disc: *E* = 4.2 MPa, ν = 0.42 (17)]. This spine model was then implemented to establish equivalent loads that would be applied to the brace during application. The equivalent loads were found to be a 3N/mm traction applied along the C curve from T7 to T9, a 34.6N force at the T1 level and a 62.5N force at T12.

**Figure 1:**
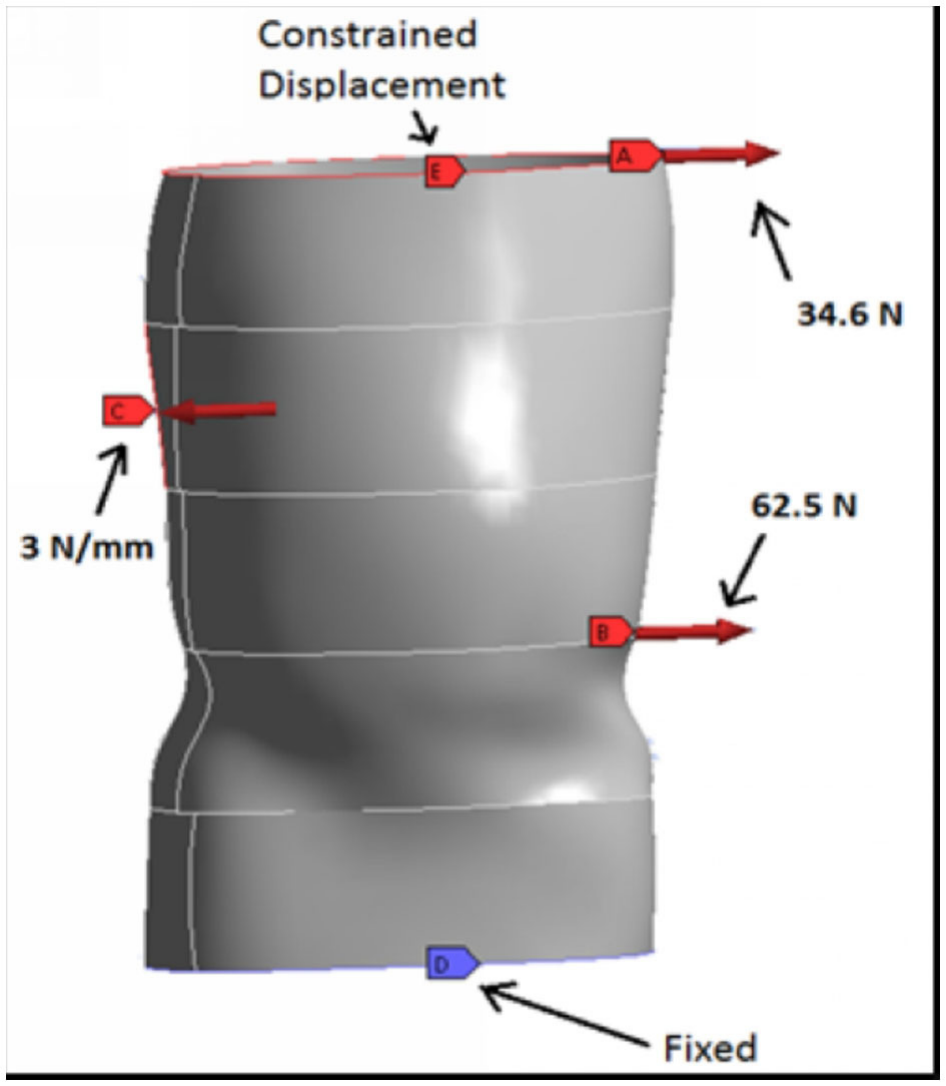
Finite element model of a patient's brace. The brace contains no cuts-outs. The results of the analysis serve as the baseline for subsequent analyses with cut-outs.

Computer Aided Design (CAD) software (SolidWorks Dassault Systems, Concord MA) was implemented to construct a CAD model of the brace. This CAD model was based on an optical scan of an existing recycled brace of typical size and geometry. It was then resized in the CAD software to fit the spine model. Using this CAD model, a separate finite element model was constructed with Ansys (Ansys, Canonsburg, PA). On this FEA model, the forces noted above were applied as well as a displacement constraint at the top and a fixed constraint at the bottom of the brace to generate a proper mechanical model of the brace (Figure 1). This finite element model had no cut outs and was used to establish a baseline analysis of the brace. For this analysis, a brace with a 2 mm thickness was used. The stress distribution as well as the deformation in the brace thickness was established and areas of low stress were identified as possible regions where the hole pattern cut-outs may be placed.

Subsequent analysis incorporated hole patterns or window cutouts. The mechanical loading of the brace is such that two criterions must be met. One criterion is that the stress cannot exceed the failure stress. The other criterion is that the deformation cannot exceed a maximal amount otherwise the reaction forces generated by the brace will not be able to stabilize the curvature. Both criterions are discussed in subsequent sections.

#### Stress Criterion

To define the structural integrity of the brace, a structural factor of safety was determined. This is a standard approach typically used in structural analysis. The standard method of the Pugsley method was used to obtain this value (18). A value of 2.32 was established. Only designs which give a facture of safety of 2.32 are considered good designs.

#### Deformation Criterion

**Figure 2:**
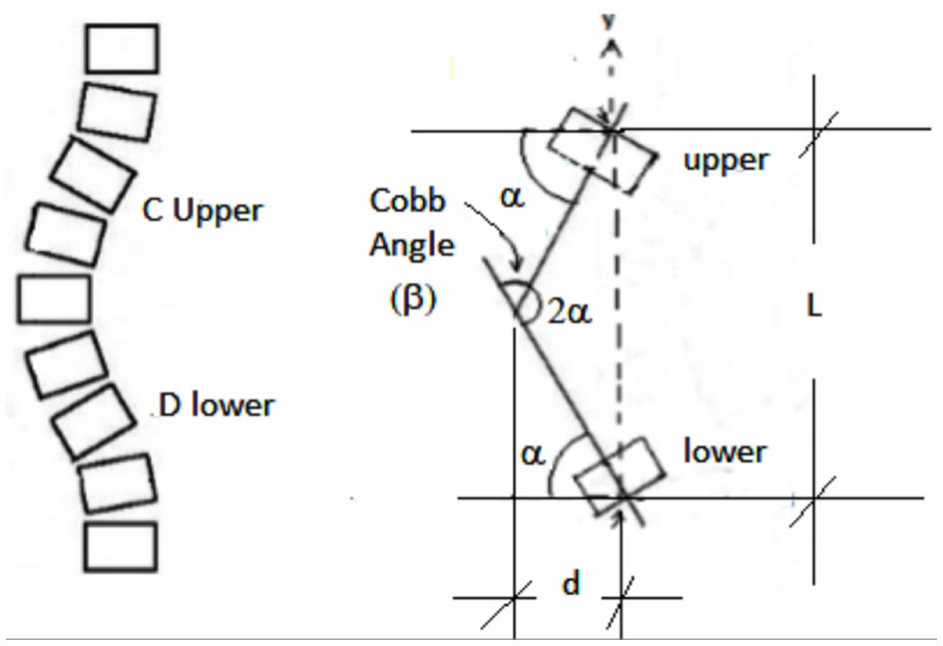
Relationship of Cobb angle to maximum lateral displacement of the spinal column/brace.

To determine the deformation criterion an examination of the mechanics of the spine and brace was undertaken. The brace applies mechanical forces to the spine using a 3 point or four-point bending mechanism. Consider Figure 2, the distance, d, from the apex of the curve to the longitudinal axis of the spine column is the key factor in generating the restorative moment. To be an effective brace, the brace must generate the proper moment to reduce this distance. If the deformation of the brace is larger than this distance, then the brace will not be able to generate the proper corrective moment.

In this analysis, the rib cage is assumed to be rigid and that forces applied to the brace are directly transferred to the spinal column. It is known that the rib cage increases the stiffness of the torso by 33% (19). Thus, the rib cage is stiffer than the spinal column with respect to lateral deflection. So, while the rib cage affects the magnitude of the restorative forces and moments, it does not affect the relationship between the applied restorative moment and the lateral deflection of the spinal column. Hence, ignoring the rib cage in the calculation will not appreciably affect the calculation of the maximum allowable deformation. Furthermore, as the amount of lateral deflection needed to restore the spinal column may be related to the Cobb angle by examining geometry in the frontal plane, the maximum deformation of the brace may also be related to the Cobb angle.

If β is the Cobb angle and using the geometry shown in Figure 2, we have that:


(1) $$\begin{array}{r} 2\alpha +\beta =180 \end{array}$$

Also, from the geometry we have that:


(2) $$\begin{array}{r} d=\frac{L}{2\tan\alpha } \end{array}$$

By substitution of Equation (1) into (2) we obtain for the deformation, d, in terms of the Cobb angle. Thus,


(3) $$\begin{array}{r} d=\frac{L}{2\tan\left(\frac{180-\beta }{2}\right)} \end{array}$$

Equation (3) may now be used to determine the maximum allowable deflection for the brace at the apex of the curve. For the spine geometry considered in the study, L was 40 mm and the Cobb Angle (β) was 22°. Employing Equation (2) we obtain for d a value of 4.9 mm. If the brace completely corrects the position of the spine to a Cobb Angle of 0°, a deformation of 4.9 mm in the lateral direction of the brace will allow the spine to return to its uncorrected position. If the goal is to stop the condition from worsening, the maximum allowable deformation with a Cobb Angle of 22° will be 4.9 mm. A deformation greater than this value indicates that the brace no longer supports the deformity and does not apply the necessary corrective forces. So, the maximum allowed deformation is 4.9 mm.

#### Brace Material Properties

Linear models for the brace were used. Thus, results would scale linearly with the mechanical properties. Furthermore, once a proper brace thickness is chosen so that the brace is a sound structural design, results obtained by introducing cut-outs to the brace are not affected by the material properties as these results are compared directly to results for a brace without any cut-outs. This ensures that general conclusions may be made without being influenced by the materials properties. However, for the simulations undertaken in the study, values from a plastic polymer from additive manufacturing (Armadillo, NinjaTek 3D, Manheim PA) was used. This material had a Secant modulus of 307.64 MPa (44.62 ksi), strength of 17.59 MPa (2.55 ksi) and a Plastic Polymer Yield Stress of 12.89 MPa (1.87 ksi). Secant Modulus and Plastic Polymer Yield Stress was used as this is standard practice when analyzing plastic polymers using linear material models.

Properties for this material was obtained by using standard tensile testing at the Milwaukee School of Engineering Mechanical Testing Laboratory with an MTS Alliance load frame. All print directions were tested. Mechanical properties where the loading is applied normal to the layering direction are typically the lowest. The primary loading in the brace is in this fashion hence these properties were used in the brace design and it is these properties that are listed above. Thus, in the design of the brace, the print direction was included in the design process.

### Hole Patterns

**Figure 3:**
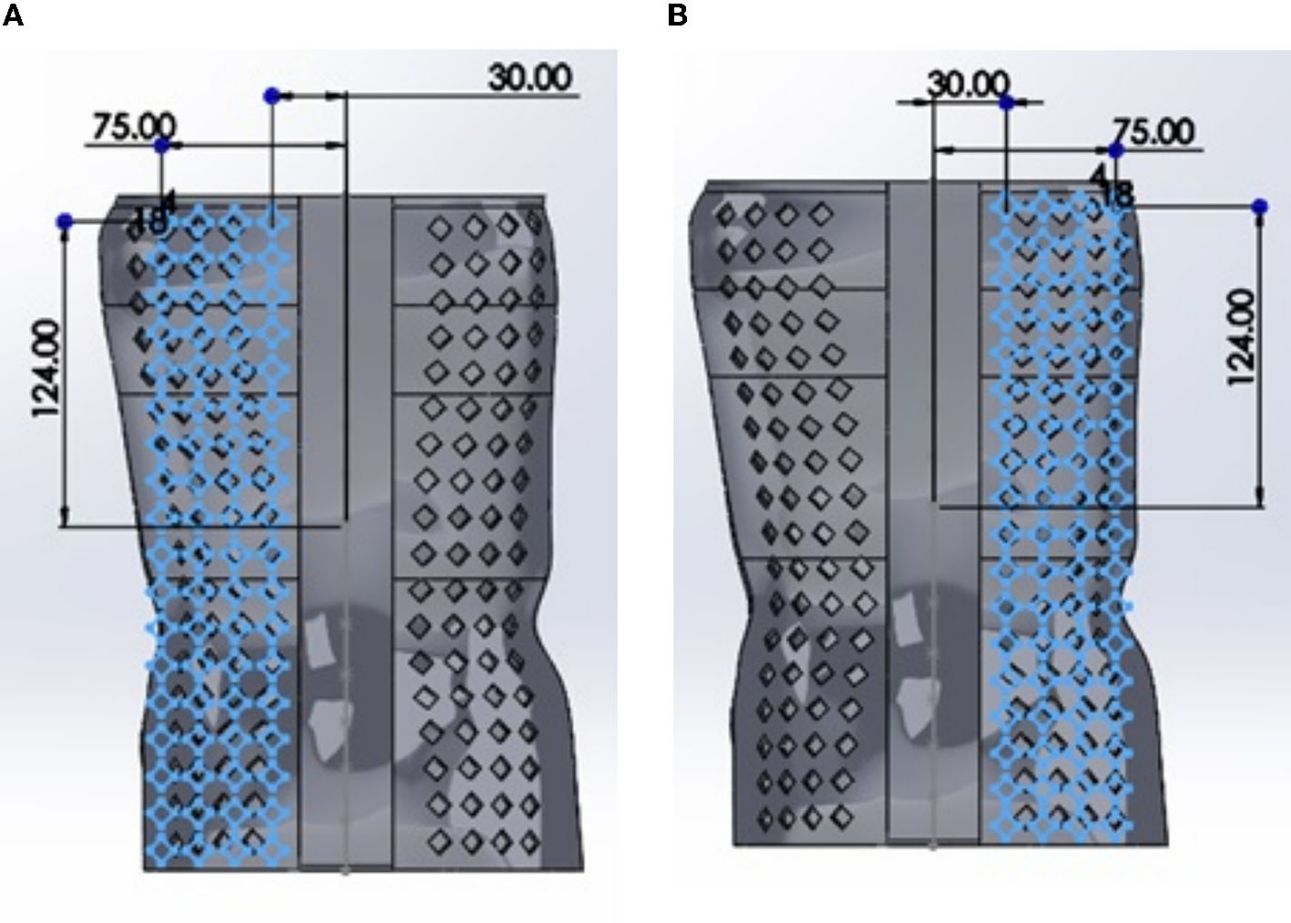
An example showing hole placement on the back of the brace with a diamond pattern. The same approach was used for the other geometries but are not shown for the sake of brevity. In **(A)**, the holes with example dimensions are shown whereas in **(B)**, the right-hand side holes are shown.

Based on the results of the baseline analysis, the hole pattern was added to the brace in locations where the stress was minimal. The hole pattern was standardized by sketching on a linear plane offset from the back of the brace (see Figure 3). The dimensions to the first hole upper (left corner) were standardized as well as the pattern array (number of rows and columns). The SolidWorks^®^ “wrap” tool was used to wrap the sketch onto the surface of the brace. This resulted in a hole pattern where each hole was perpendicular to the surface of the brace.

To determine the optimal hole shape, the shape of the hole was changed and all other parameters relating to the sizing, spacing and location of the pattern were kept constant. To achieve constant parameters allowing for a valid comparison to be made, all hole shapes were set with a basic area defined by a hole having an equivalent diameter (D).

Initially, a 10 mm equivalent diameter (D) was used along with a spacing between the holes (L) of 15 mm to give L/D=1.5. The finite element analysis was performed for each hole pattern geometry using this ratio. The factor of safety, volume removed by the hole pattern, peak stress and peak deformation were recorded. In subsequent simulations the L/D ratio was varied to investigate the relationship between hole geometry, factor of safety, peak stress and deformation until an optimal hole shape was identified relative to these parameters.

With the optimal hole shape identified, the next step was to determine the ideal size of holes and optimal spacing between them. To accomplish this, multiple iterations of FEA simulations were performed. The parameters used to determine the ideal pattern are as follows: percent volume removed, L/D ratio (where L is the center to center spacing and D is the equivalent diameter) and the factor of safety. Based on the results from the previous simulations, these iterations were only carried out for the hexagonal pattern as this proved to be the optimal shape.

### Windows

#### Abdomen Window

**Figure 4:**
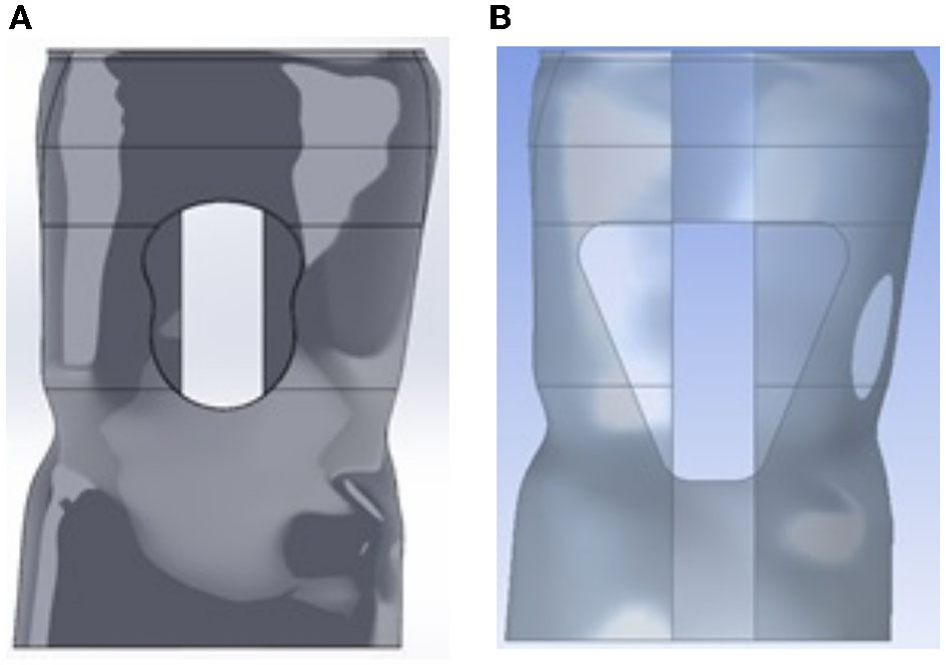
Different shape design for the abdominal window. **(A)** “Bib” design for abdominal window while **(B)** Trapezoidal design.

The abdomen window was placed at the center of the abdomen (see Figure 4). Therefore, there was no need to undertake an analysis of the location of the window. Only, the effect of the size and shape of the abdomen window on the brace was considered. Analyzes were carried out by using Ansys^®^ and the original solid brace design with varying shapes for the abdomen window. The shapes considered were circular, trapezoidal and “bib” shape. These geometries are widely used in practice. For each simulation, the cut out was added to the solid brace model and analyzed using Ansys. In subsequent simulations, the size of the window was increased, and the maximum stress and deformation recorded. The process was repeated for each window geometry.

#### De-rotation Window

**Figure 5:**
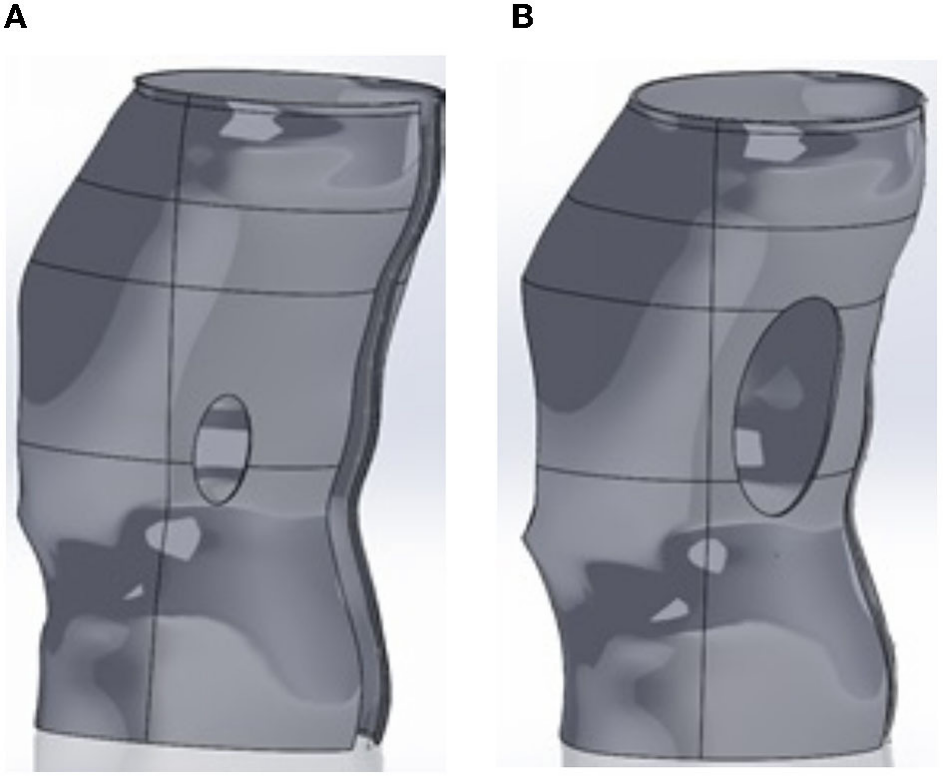
CAD model of the brace with added oval shaped de-rotation window. Two examples are given showing variation in window size. The window is smaller in **(A)** than in **(B)**.

**Figure 6:**
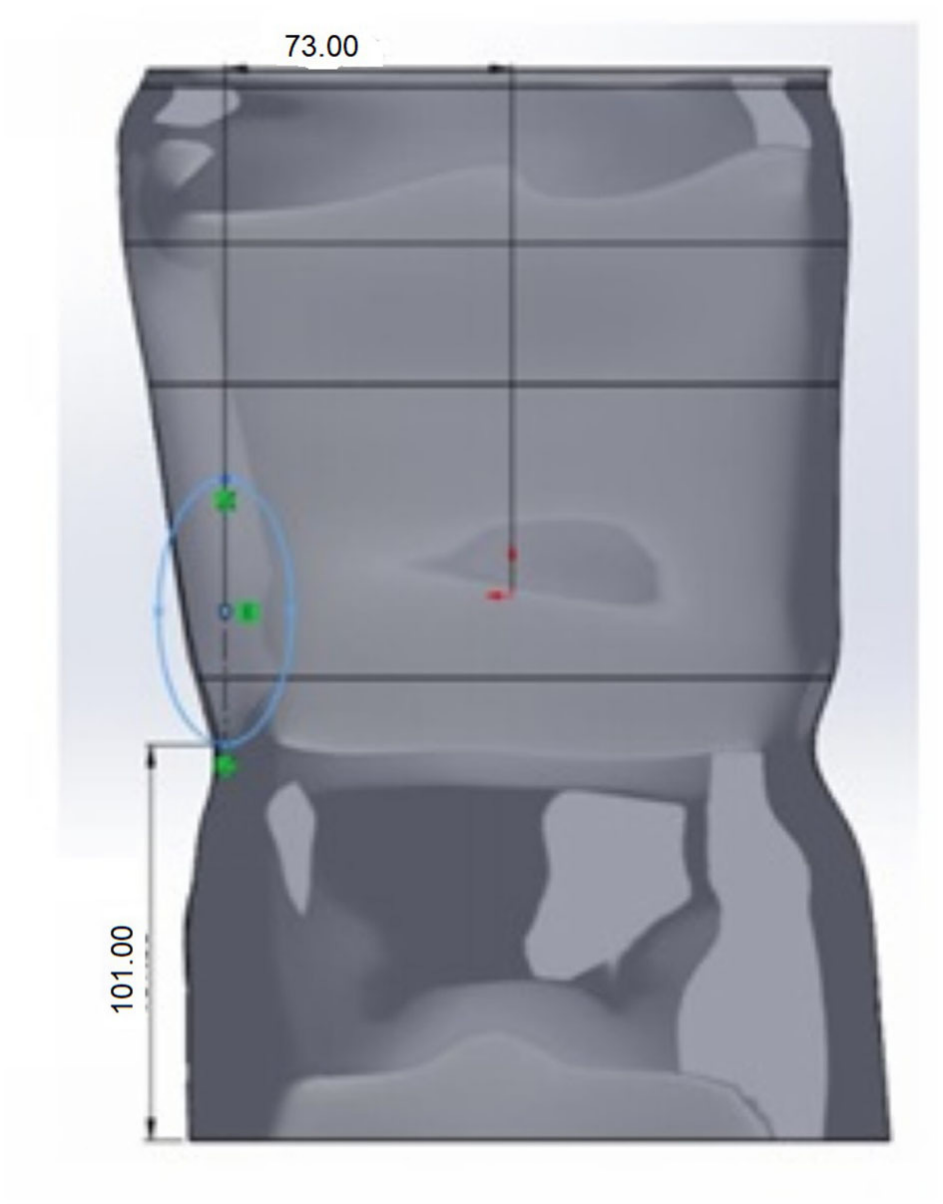
Though positioned posteriorly and so that the center is 1–2 vertebrae below the apex of the C curve, the derotation window position was varied with respect to the base of the brace and the medial line.

The de-rotation window had an elongated oval shape or a shape resembling the outline of a boot. The window center was placed roughly 1-2 vertebrae below the apex of the C curve and partially on the lateral but primarily posterior aspect (see Figure 5). In the simulations using Ansys^®^, the size of this window and location was varied. However, in placement of the window it was positioned so that the center of the window was positioned as noted above. Position variation was defined using a distance from the base of the brace and a distance from the medial line as shown in Figure 6.

## Results

### Hole Patterns

Table 1 shows the results from examining the various hole patterns. We see that all hole patterns have a factor safety greater than two 2.32. The hole shapes remove differing amounts of material and both the peak stress and peak deformation vary depending on the shape. The shape which removes the most amount of material and generates a factor of safety which is closest to the desired 2.32 factor of safety is the hexagon geometry.

**Table 1:** Results obtained with constant size and spacing but varying hole geometry.

**Hole**	**Factor**	**Volume**	**Peak**	**Peak**
**shape**	**of Safety**	**removed**	**stress (MPa)**	**Peak deformation**
		**(cm^**3**^)**		**(mm)**
None	4.82	0.00	2.86	7.37
Circle	2.81	30.28	4.90	5.50
Triangle	3.92	12.34	3.52	2.53
Diamond	3.06	19.43	4.50	2.70
Hexagon	2.62	24.80	5.26	2.82

In Table 2, we show the results obtained from the FEA simulations when hole spacing, equivalent diameter, and L/D ratio are varied. Because of the results shown in Table 1, only the hexagonal pattern was investigated. The results indicate that an equivalent diameter of 10 mm, an *L*/*D* ratio of 1.2 and spacing of 12 mm gives a factor safety of 2.32. We also see that the peak deformation in this scenario is 4.61 mm, which is less than the maximum allowed value of 4.90 mm therefore this is the optimal hole size and spacing scenario.

**Table 2:** Effect of varying L/D ratio, spacing, and equivalent diameter on the stress, factor of safety and volume removed.

**L/D ratio**	**Spacing (mm)**	**Equivalent diameter (mm)**	**Maximum stress (MPa)**	**Maximum deformation**	**Factor of safety**	**% Volume removed**
1.20	12.00	10.0	5.94	4.61	2.32	10.09
	18.00	18.00	6.48	4.82	2.13	11.48
1.25	6.25	5.00	5.10	4.62	2.70	9.21
	12.50	10.0	5.55	4.38	2.49	9.23
	18.75	15.0	6.79	4.71	2.04	10.82
1.50	7.50	5.00	5.00	4.56	2.77	5.95
	11.25	7.50	4.32	4.20	3.20	6.26
	15.00	10.0	6.10	4.40	2.27	6.98
	22.50	15.0	6.25	4.30	2.20	6.74
2.00	10.00	5.00	5.22	4.01	2.69	3.81
	20.00	10.00	4.91	4.00	2.80	3.82
	25.00	12.50	6.12	3.93	2.25	3.88
	30.00	15.00	7.04	4.08	1.96	4.80

*The target is to find a design which gives a factor of safety of 2.32 and a deformation < 4.90 mm*.

### Windows

#### Abdomen Window

The circular shape was observed to cause high stresses in the brace though the variation in the size of the window did not impact the factor of safety drastically. The deformation increased significantly as more volume was removed but it remained under the maximum 4.5 mm deformation at the location of the loading even for an oversized window. Even for a small opening with 2.17% volume of material removed, the factor of safety was determined to be under the required value.

The rest of the study focused on the trapezoidal and “bib” shape. From the simulations, it was observed that the targeted factor of safety and deformation was attained even with significant window opening size (up to 10%).

**Figure 7:**
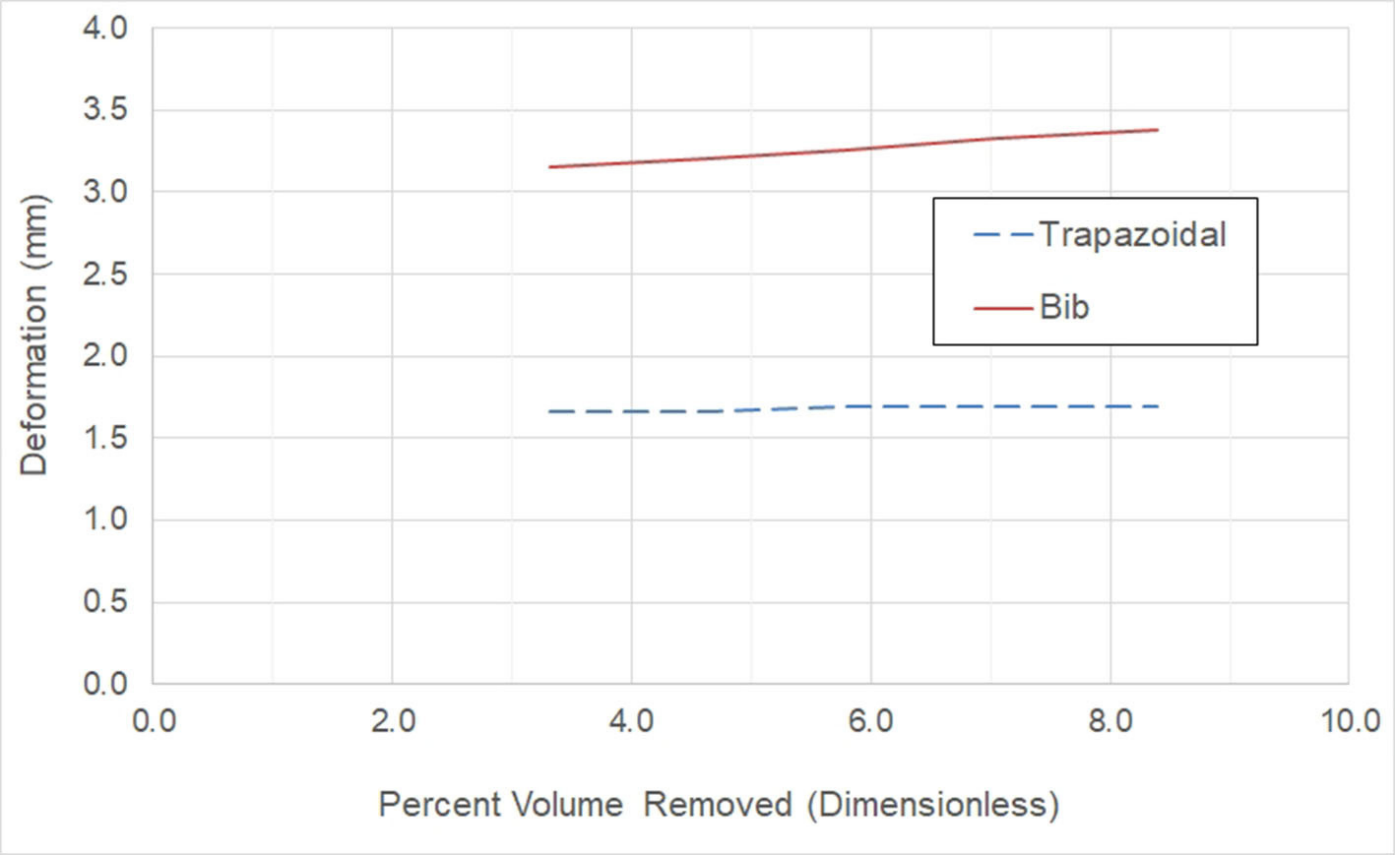
Effect on the deformation for the trapezoidal and bib window geometry as the abdomen window size increases.

Figure 7 illustrates the effect of window size on the deformation for the two window geometries under consideration. We see that there is little effect on the deformation for the trapezoidal shape, but the deformation increases as the window size increases for the “bib” geometry. With either window geometry, the deformation is below the maximum value of 4.90 mm.

**Figure 8:**
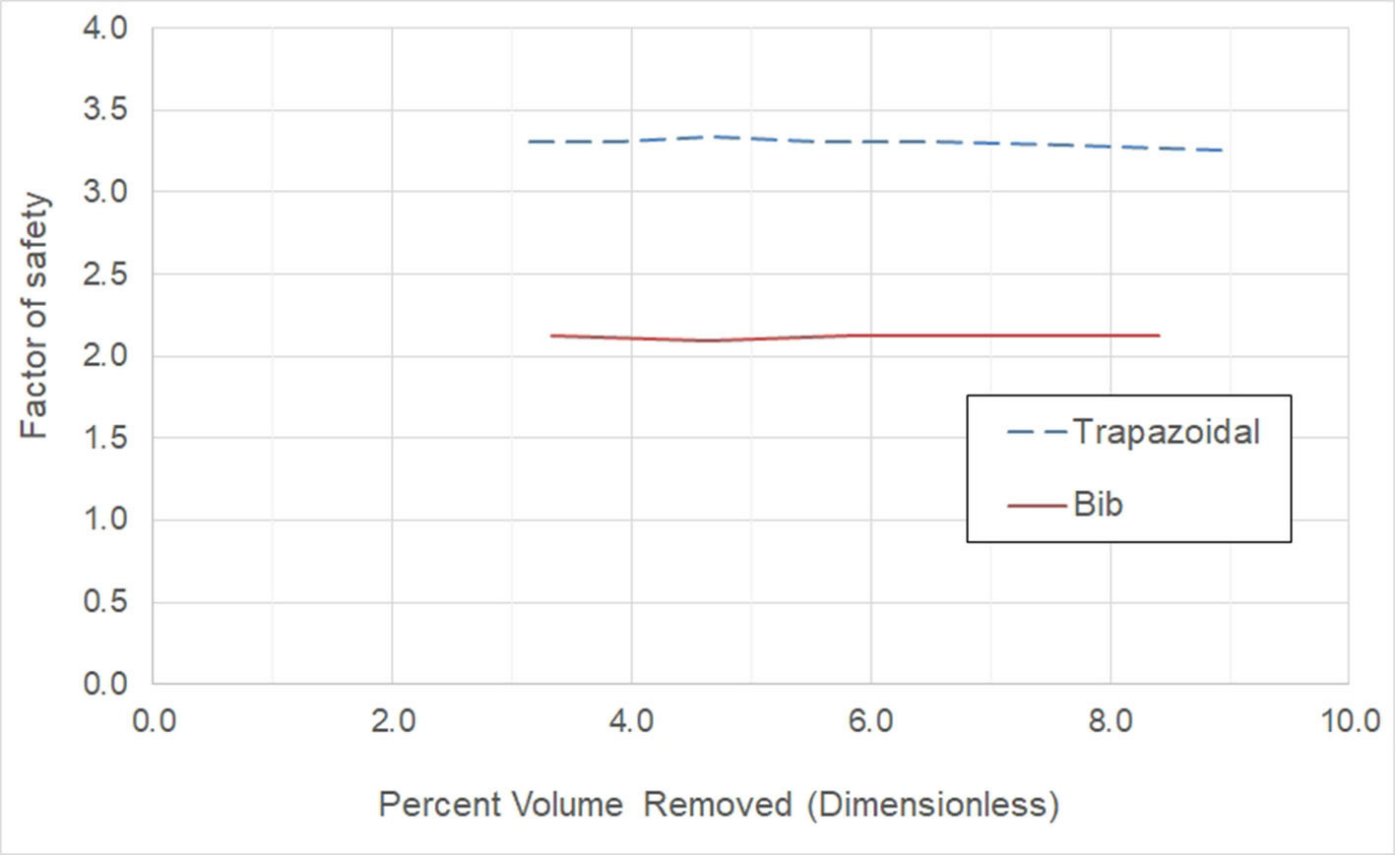
Examination of the effect on the factor of safety as the abdomen window size increases.

Figure 8 shows the effect on the factor of safety by a variation in window size. We see that there is no design for the trapezoidal window shape which will lead to the required factor safety of 2.32. The “bib” shape on the other hand attains this goal. In either case, there is very little effect on the factor safety as the window size increases.

#### De-rotation Window

Table 3 illustrates the effect on the safety factor and deformation due to an increasing size of the derotation window. From the table, we see that there is little to no effect on the safety factor and deformation due to a variation in size of the window.

**Table 3:** Effect on the factor of safety and maximum brace deformation as the size of the derotation window is increased.

**Window size**		**Factor of safety**	**Maximum** **deformation (mm)**
**Height (mm)**	**Width (mm)**		
24	12	2.11	3.32
30	15	2.12	3.32
34	17	2.12	3.32
40	20	2.11	3.32
44	22	2.12	3.32
50	25	2.11	3.33

Table 4 shows the effect on the factor of safety and maximum deformation as the height from the bottom (base) is increased (also see Figure 6). The window placement is controlled relative to the base of the brace. With the placement moving superiorly as the distance to the base increases.

**Table 4:** Effect on the factor of safety and maximum deformation as the location of the derotation window is changed.

**Distance from**	**Distance from**	**Factor**	**Maximum**
**base (mm)**	**brace medial line (mm)**	**of safety**	**deformation (mm)**
101	73	2.12	3.32
111	74	2.11	3.31
121	75	2.11	3.31
131	76	2.12	3.31
141	77	2.12	3.31
151	78	2.12	3.31

## Discussion

### Hole Patterns

Table 1 indicates that hexagonal pattern is the optimal hole pattern. This pattern removes the most volume of material, leads to a factor safety closest to 2.32 and generates a deformation <4.90 mm and hence satisfies the stress and deformation criterions. So, the addition of the hole pattern to the brace would not adversely affect the structural integrity of the brace. Furthermore, by examining Table 2 we see that an optimal hexagonal hole size and spacing is obtained when the equivalent diameter of hexagonal hole is10 mm and the spacing between the holes is 12 mm. It is interesting to note that 10.09% of the volume of the brace is removed by this pattern. This would reduce the weight of the brace by this amount.

In the analysis for spacing of the holes, linear patterns were considered as well as staggered patterns. Linear patterns are patterns where the holes are aligned. Whereas staggered patterns are such that the holes are offset from one another. The simulations showed that none of the linear patterns generated a factor of safety of at least 2.32. This indicates that linear patterns are not acceptable in the design.

A brace design based on a specific patient was used for the simulations. However, the conclusions of the study are not limited by using data from a specific patient. This is because while the brace size and applied forces may change from one patient to another, the variation will be linear. For example, an increase in applied force will change the minimum required brace thickness and magnitude of the stress distribution, it will not change the location of the maximum deformation or the stress distribution. The thickness will scale linearly with the increase in applied forces. So, the observation of which pattern is more optimal compared to the others will not be impacted. Since the thickness scales linearly with the applied forces, the increase in material thickness will lead to increase in the material available to carry the applied forces. This implies the hole size spacing and pattern of distribution will not be affected.

### Windows

From the simulations, even a small window size led to a design where the factor of safety requirement could not be met if the abdomen window had a circular shape. This indicates that the circular abdominal shape is not a valid shape for use in braces. Conversations with orthotists regarding this circular shaped abdomen window indicated that there is an effect on patient comfort and the patient may be susceptible to regurgitation [4, 21]. Thus, the circular shaped abdominal window is not suitable for use in braces from the structural as well as clinical aspect.

Examining Figures 7, 8, we conclude that while both the trapezoidal and “bib” abdomen window shapes lead to designs where the deformation requirement is met, only the “bib” design allows the target goal of a 2.32 factor of safety to be achieved. Thus, the “bib” design is the only abdominal window geometry that can achieve both the deformation and factor of safety requirement. Furthermore, in conversations with orthotists, clinical practice has shown that the trapezoidal shape may lead to regurgitation [4, 21]. Thus, only the “bib” geometry should be used for the abdominal window. As there is little effect on the size on the deformation and factor of safety due to the size of the window, the criterion for sizing this window is not controlled by structural needs rather it is controlled by the clinical need. Thus, the abdomen window should be sized to fit the patient's abdomen.

For the derotation window, the simulations indicate that there is no effect on the deformation or factor of safety due to the placement of the window or the size of the window. This is because relatively speaking the window is located far from the location of the applied brace loads. Thus, the size and location of the window should be based on clinical need rather than a structural need. The size of the window should be such that it allows for the proper derotation of the spine. The location should be 1–2 vertebrae below the apex of the C curve, lateral but slightly posterior.

## Conclusions

For a sound structural brace design with no impact on the functionality of the brace, a hexagonal hole pattern should be used with a spacing of 12 mm and equivalent hole diameter of 10 mm. This indicates that each hole in the pattern should have a surface area of 78.54 mm^2^.

Windows in the abdominal area should be of “bib” shape. This shape does not impact the structural efficiency of the brace and prevents regurgitation by the patient. As window size does not affect the stress and deformation significantly, the size of this window should be based on the minimum required size needed to permit the patient to breathe comfortably.

For the derotation window, there is no effect on the structural integrity due to the size or shape of this window and its implementation should be based on clinical need to allow for proper derotation of the spine.

## Limitations

Geometry from only one case from the literature was used in the study. While the results may be scaled for larger patients due to the linearity of the analysis, the plan is to consider additional patient geometries in the future.

## Data Availability Statement

The raw data supporting the conclusions of this article will be made available by the authors, without undue reservation.

## Author Contributions

All authors listed have made a substantial, direct, and intellectual contribution to the work and approved it for publication.

## Conflict of Interest

The authors declare that the research was conducted in the absence of any commercial or financial relationships that could be construed as a potential conflict of interest.

## Publisher's Note

All claims expressed in this article are solely those of the authors and do not necessarily represent those of their affiliated organizations, or those of the publisher, the editors and the reviewers. Any product that may be evaluated in this article, or claim that may be made by its manufacturer, is not guaranteed or endorsed by the publisher.
